# Spectroscopic detection of halogen bonding resolves dye regeneration in the dye-sensitized solar cell

**DOI:** 10.1038/s41467-017-01726-7

**Published:** 2017-11-24

**Authors:** Fraser G. L. Parlane, Chantal Mustoe, Cameron W. Kellett, Sarah J. Simon, Wesley B. Swords, Gerald J. Meyer, Pierre Kennepohl, Curtis P. Berlinguette

**Affiliations:** 1 Department of Chemistry, The University of British Columbia, 2036 Main Mall, Vancouver, BC Canada V6T 1Z1; 2 Stewart Blusson Quantum Matter Institute, The University of British Columbia, 2355 East Mall, Vancouver, BC Canada V6T 1Z4; 3 Department of Chemistry, The University of North Carolina at Chapel Hill, Murray Hall 2202B, Chapel Hill, NC 27599-3290 USA; 4 Department of Chemical & Biological Engineering, The University of British Columbia, 2036 East Mall, Vancouver, BC Canada V6T 1Z3

## Abstract

The interactions between a surface-adsorbed dye and a soluble redox-active electrolyte species in the dye-sensitized solar cell has a significant impact on the rate of regeneration of photo-oxidized dye molecules and open-circuit voltage of the device. Dyes must therefore be designed to encourage these interfacial interactions, but experimentally resolving how such weak interactions affect electron transfer is challenging. Herein, we use X-ray absorption spectroscopy to confirm halogen bonding can exist at the dye-electrolyte interface. Using a known series of triphenylamine-based dyes bearing halogen substituents geometrically positioned for reaction with halides in solution, halogen bonding was detected only in cases where brominated and iodinated dyes were photo-oxidized. This result implies that weak intermolecular interactions between photo-oxidized dyes and the electrolyte can impact device photovoltages. This result was unexpected considering the low concentration of oxidized dyes (less than 1 in 100,000) under full solar illumination.

## Abstract (web summary)

Dye-sensitized solar cells rely on molecular dyes to absorb light and conduct electrons. Parlane et al. show that weak forces such as hydrogen bonding can be responsible for the dye regeneration step of solar cells and have an impact on the photovoltage and the efficiency.

## Introduction

Dye-sensitized solar cells (DSSCs) rely on molecular dyes fixed to a mesoporous semiconductor film to convert sunlight into electricity^1,2^. The efficiency of these devices is strongly dependent on the relative rates of dye regeneration and several deleterious interfacial charge recombination reactions. The dye regeneration step is an interfacial electron transfer reaction between an immobilized oxidized dye and a soluble iodide species^2^. It can therefore be assumed that the rate constant of the regeneration step, *k*_reg_, can be correlated to the extent of the interactions between the dye and the electrolyte^3,4^.

Computational studies and indirect experimental evidence point to the reactive portion of the dye as the electron-rich donor unit, where the lowest unoccupied *β*-spin orbital (*β*-LUSO) resides following photo-oxidation of the dye. For example, density functional theory (DFT) and natural population analyses (NPAs) of ruthenium and organic dyes have shown that regeneration is preceded by formation of an ionic dye^+^-iodide pair^5,6^. A hypsochromic shift of the metal-to-ligand charge transfer (MLCT) band, changes in ligand vibrational modes upon exposure to iodine^7,8^, and single-crystal X-ray structural data collectively support a dye-iodine interaction^9^ but do not directly report on the dye regeneration step that involves an oxidized dye and I^−^. The strongest experimental evidence for a dye^+^-iodide adduct is arguably the possible detection of a transient species by nanosecond laser spectroscopy^10^, and our previous studies outlining how changing donor unit constituents can affect *k*_reg_^3,4^. We also demonstrated that differences in *k*_reg_ impacted the measured photovoltages for DSSCs^3,11^.

Our most recent work on this subject has sought to leverage halogen bonding between the dye and the electrolyte as a means to enhance device performance^4,11^. A halogen bond is an interaction between a Lewis base and a covalently bound, polarizable halogen that acts as an electrophile. The origin of this effect is an anisotropic distribution of the electron density around the halogen substituent, forming an electropositive region known as the *σ*-hole^12,13^. The size of the *σ*-hole is negligible for fluorine, but increases with increasingly polarizable halogens as well as the electron-withdrawing strength of the covalently bound functional groups. We previously tested the utility of halogen bonding in the DSSC by synthesizing a homologous series of donor-π-acceptor dyes that differed only in the identity of the halogen substituents on the triphenylamine (TPA) donor portion of each molecule (Dye X, where X is F, Cl, Br, or I)^4,11^. In our original studies, it was found that *k*_reg_ increased in direct proportion to the polarizability of the halogen substituents, and that this increased regeneration rate correlated with an improved device performance for Dye-I. While these results were consistent with halogen bonding being operative, we could not conclusively rule out other intermolecular interactions such as London dispersion forces as being responsible for the measured effects. Moreover, accepted dogma does not support the notion of halogen bonding with these dyes because the carbon atoms that the halogen substituents are attached to are not sufficiently electron-withdrawing. More meaningful mechanistic information on how halogen bonding is potentially affecting electron transfer rates is therefore needed.

In this current work, we seek to address these issues by using advanced physical methods to directly observe these halogen bonding interactions in both the neutral and oxidized dyes. Halogen bonds are most commonly studied through microwave, NMR, and IR spectroscopie, as well as X-ray crystallography^14^. These techniques, however, are poorly suited to study halogen bonding at solid-liquid interfaces. Recently, Kennepohl and Beer have demonstrated the utility of K-edge X-ray absorption spectroscopy (XAS) to directly observe halogen bond formation in solution^15^. Here, we have adapted this technique to observe electrolyte interactions with Dye-X absorbed to mesoporous TiO_2_ films, using chloride as a surrogate for the iodide electrolyte. These XAS measurements clearly show a spectroscopic signature corresponding to halogen bonding between the electrolyte and oxidized Dye-X^•+^ but not with neutral Dye-X. In light of our previous work, this data strongly suggests that the dye regeneration step is affected by halogen bonding with the oxidized dye and only the oxidized dye—a surprising observation that reconciles other lines of experimental evidence^3,5,8,10,16,17^. These results offer unprecedented insight into the spatial and temporal binding characteristics between the electrolyte and the oxidized dye.

## Results

### Characteristics of the Dye-X compound series

**Fig. 1:**
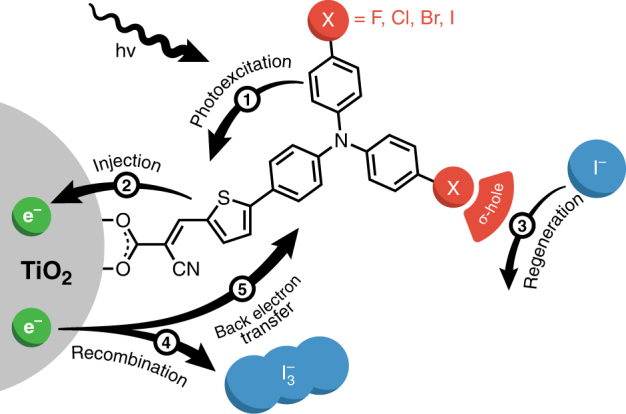
Dye structure and an overview of interfacial electron transfer reactions. Molecular representation of Dye-X on a titanium dioxide (TiO_2_) particle (not drawn to scale) where different halogen substituents (X = F, Cl, Br, or I) are bound to the TPA portion of the dye. The carboxylate group serves to fix the dye to the TiO_2_ nanoparticle and directs the halogen substituents towards the soluble nucleophilic species (e.g., iodide)

The structures of the Dye-X series are presented in Fig. 1, and the synthesis, optical, and redox properties of these compounds have been reported previously^4,11^. Cyclic voltammograms recorded on the dyes immobilized on TiO_2_ produced quasi-reversible Dye-X^•+/0^ redox couples that deviated by merely 60 mV for the series. The absorption spectra recorded on Dye-X in solution and when immobilized on TiO_2_ also showed only nominal changes in absorption profiles across the series, with absorption band maxima centered at *ca*. 430 nm and 450 nm, respectively. These experiments indicate that the identity of the halogen atom on the dye does not significantly influence the electronic structure of the molecule, thereby enabling the interfacial reactivity to be quantified while holding Δ*G*° for the interfacial reactions at parity.

### X-ray absorption spectra

**Fig. 2:**
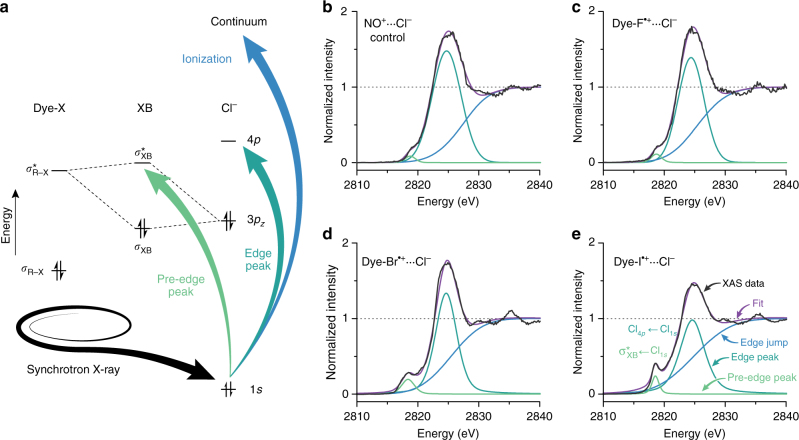
Chlorine K-edge XAS spectra of the Dye-X series. **a** Schematic of molecular orbitals involved in halogen bonding and allowed transitions upon excitation of the Cl_1s_ electron by synchrotron X-ray radiation. **b**–**e** Chlorine K-edge X-ray absorption spectroscopy (XAS) spectra of the Dye-X···Cl^−^ series. Spectra were modeled with BlueprintXAS peak fitting analysis and the resulting components of each fit are shown with each spectrum

To resolve the existence of an interfacial halogen bond, we set out to use XAS as a tool to directly measure the electronic coupling (or orbital overlap) between the dye and the electrolyte species. Chlorine K-edge XAS is capable of measuring the degree of covalency in metal−chlorido bonds^18–20^ and charge transfer in donor–acceptor systems, including halogen bonds involving a chloride electron donor^15, 21^. Charge transfer from the chloride to a halogen bond donor is detected by a lower-energy, pre-edge feature in the chlorine K-edge XAS spectrum that arises from charge depletion of the filled chlorine 3p manifold (Fig. 2). The integrated intensity of this pre-edge feature is directly proportional to the degree of mixing between the Cl_3p_ orbital and the electron-accepting orbital, thus providing the means to identify and quantify a halogen bond^18–20^. Chloride is an effective surrogate for the iodide species used in the DSSC because the chlorine K-edge is at an accessible energy, the nucleophilicity of chloride is comparable with iodide^22^, and longer timescales are available for measuring halogen bond interactions with the oxidized dyes given that the reduction of Dye-X^•+^ by chloride is not favorable (*E*° = 1.36 V vs. NHE for Cl_2_ + 2e^−^ → 2Cl^−^)^23,24^.

Chlorine K-edge XAS data was recorded on a sensitized, mesoporous TiO_2_ film deposited on glass. Each film was stained with Dye-F, Dye-Br, or Dye-I, then sealed with X-ray-transparent polypropylene before being filled with an acetonitrile solution of 100 mM tetrabutylammonium chloride. The oxidized forms of the dyes on TiO_2_ were obtained via chemical oxidation by washing the films with a saturated solution of nitrosonium tetrafluoroborate in acetonitrile prior to filling with electrolyte. To account for possible chloride-nitrosonium interactions, an undyed film was also washed with nitrosonium tetrafluoroborate before filling with electrolyte to serve as a control (See Supplementary Fig. 4). K-edge XAS spectra are dominated by electric dipole-allowed p ← s transitions (Δℓ = ± 1). At the chlorine K-edge, the edge features are dominated by Cl *n*p ← 1 s excitations where *n* ≥ 4; the intense white line feature resulting from the lowest energy transition of this type (Fig. 2a, turquoise)^18^. Above the K-edge, excitation results in ionization of the chlorine 1s electron to vacuum (Cl^−^ → Cl^•^ + e^−^; Fig. 2a, blue). When the chloride forms an adduct with the dye, Dye-X^•+/0^···Cl^−^, orbital overlap of a filled Cl_3p_ orbital with the antibonding carbon-halogen bond (σ*_R–X_) results in charge transfer from Cl^−^ → σ*_R–X_ driving formation of an empty orbital with Cl_3p_ character (σ*_XB_), and thus a new allowed transition. This new transition is at a distinctively lower energy than the 4p ←1s transition (Fig. 2a; green).

**Fig. 3:**
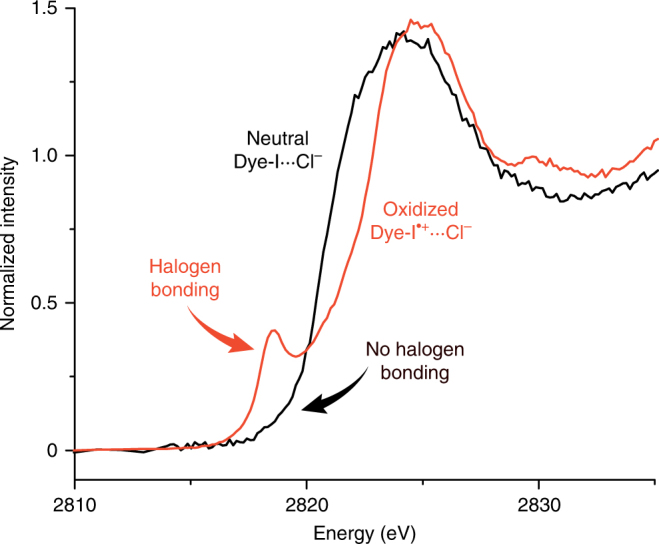
XAS spectra of neutral and oxidized dyes in presence of chloride. Chlorine K-edge X-ray absorption spectroscopy (XAS) spectra recorded on TiO_2_ substrates stained with Dye-I and Dye-I^+^ in the presence of 100 mM acetonitrile solution of tetrabutylammonium chloride. The pre-edge feature at *ca*. 2818 eV is indicative of a halogen bonding interaction where charge is transferred from chloride to the σ*_XB_

The XAS spectra recorded on the Dye-X series on TiO_2_ did not show a pre-edge feature (Fig. 3; black) due to very poor orbital overlap or the absence of halogen bonding. Both possibilities lead to the same interpretation that minimal halogen bonding occurs between the neutral dyes and the electrolyte. A poor interaction between Cl_3p_ and the σ*_R–X_ may be due to the σ*_R–X_ being significantly higher in energy than the Cl_3p_ electron donor, or the σ*_R–X_ being localized more towards the carbon rather than the halogen substituent. Previous halogen bonding experiments have shown the importance of placing strong electron-withdrawing substituents around the halogen bond donor to realize the *σ*-hole, particularly when in solution^25^. On these grounds, it is anticipated that Dye-X would not participate in a halogen bond significantly enough to result in a detectable XAS signal due to their lack of electron-withdrawing units in proximity to the halogen. Upon oxidation of the dye, the σ*_R–X_ will be lowered in energy and the increase in polarization of the carbon-halogen bond will cause the σ*_R–X_ to be more localized towards the halogen. This scenario is more compatible with a halogen bonding interaction, resulting in a new dipole-allowed σ*_XB_ ← Cl_1s_ transition. The Dye-X^•+^···Cl^−^ series does indeed exhibit this feature at *ca*. 2818 eV (Fig. 2b–e). (Note that Chlorine K-edge XAS data is not presented for Dye-Cl, which yields a combination of features from both the electrolyte and the dye that renders analysis significantly more complex. Supplementary Fig. 3.)

Two distinct mechanisms are responsible for the pre-edge features that arise upon oxidation of the surface-adsorbed dyes. Firstly, the oxidation of the TiO_2_ surface (with NOBF_4_) generates defect sites that result in some covalent binding of halide ions to the surface (Ti ← Cl^−^) as observed in the control experiment (TiO_2_^ox^ ← Cl^−^). Secondly, chloride ions may also bind to the oxidized dye via halogen bonding (Dye-X^•+^ ← Cl^−^). We observe two pre-edge features in the Cl K-edge spectrum for Dye-Br^•+^ ← Cl^−^, a smaller feature at higher energy corresponding to defect sites (as in TiO_2_^ox^ ← Cl^−^), and a larger feature at lower energy that only occurs in the presence of the oxidized dye. The relative contributions of each feature are illustrated in Supplementary Fig. 2. We can thus estimate the magnitude of the XB interaction by subtracting contributions from the defect sites. The integrated intensity of the observed pre-edge features was determined by simultaneous peak fitting of the pre-edge and edge regions of each spectrum (Fig. 2b–e) to quantify the overall pre-edge intensity^25–^. Given the baseline intensity observed in the TiO_2_^ox^ ← Cl^−^ control, we may then estimate the contribution to the pre-edge that results from XB interactions. At this point, it is worthwhile to consider that the observed pre-edge intensity depends on two factors: the degree of covalency in the XB interactions as well as the quantity of interactions. XAS is unable to distinguish between these two effects; however, interactions with greater covalency will result in stronger interactions, which will in turn result in a greater number of interactions; these factors can therefore be considered to have a synergistic effect, enhancing the differences in the Dye-X^•+^ series. We note that the pre-edge intensity from XB interactions is essentially zero in Dye-F^•+^, and quite significant in both Dye-Br^•+^ and Dye-I^•+^. This experimental trend matches the behavior observed in our computational modeling, although a quantitative comparison is not feasible.

### Computational modeling

In order to determine the degree to which these XAS results with chloride can be generalized to the iodide-based experiments that were the subject of our previous work^4,11^, two DFT models (denoted here as methods A and B; see computational methods for full descriptions) were prepared using two different methodological frameworks to compare the thermodynamics of these interactions with the two different anions. These methodologies were based off of previously reported computational studies that have proven effective in describing halogen bonding^15, 21, 28–30^. The relevant bond distances, interaction energies, and natural bond orbital analysis results are summarized in Supplementary Tables 8–10. These results, illustrated in Supplementary Fig. 7, show the same trend in halogen bond strength across the entire dye series. Furthermore, in the case of method A, these results show nearly the same magnitude in interaction energy, regardless of whether the anion is chloride or iodide. This result supports the notion that these halogen bonds are largely agnostic to the identity of the halide nucleophile. Results with method B showed considerably larger overall magnitudes compared with method A, as well as a larger difference between chloride and iodide values. These differences can be attributed to artificial destabilization of the free ions compared to the ion complexes resulting from the absence of a solvation model in these calculations, and do not change the conclusion that the energetic trends between chloride and iodide interaction are not dependent on the anion. TD-DFT calculations based on these interaction models do not, however, adequately predict our XAS results for the Dye-X^•+^···Cl^−^ series. Exploration of a range of computational models suggest that, even though the energetics of these systems are largely insensitive to the functional and basis set utilized, the TD-DFT results are highly sensitive to the choice of computational model. The degree of charge delocalization varies quite significantly in our computational studies (see Supplementary Fig. 6 for details), and the most important factors appeared to be the degree of Hartree–Fock exchange in the applied functional, the choice of diffuse functions in the basis set, and the choice of solvation model. In-depth computational studies to resolve these issues are currently ongoing. Nonetheless, these theoretical investigations demonstrate the same energetic trends independent of the identity of the anion and the computational model employed. This observation supports the notion that the study of Dye-X^•+^···Cl^−^ interactions can be extended in a qualitative sense to the Dye-X^•+^···I^−^ interactions relevant to DSSCs.

## Discussion

As illustrated in Fig. 1 for a common TPA dye, the absorption of a photon by the dye, Eq. (1), enables fast charge injection from the excited state (Dye*) into the conduction band of a titanium dioxide (TiO_2_) semiconductor, Eq. (2). The photo-oxidized dye (Dye^•+^) is, in turn, regenerated by a reduced redox mediator (e.g., iodide), as in Eq. (3), that gets regenerated at the counter-electrode. The dye regeneration step, Eq. (3), needs to proceed more efficiently than the recombination reactions where the electron injected into the semiconductor, TiO_2_(e^−^), reacts with the oxidized mediator species triiodide, Eq. (4), or oxidized dyes on the surface, Eq. (5), in order to extract electrical work from the cell. It is therefore imperative to better understand the interfacial dye regeneration step in the collective pursuit of more efficient DSSCs.

Photoexcitation:

1 $${\mathrm{Dye}}/{\mathrm{TiO}}_2 + {\mathrm{photon}} \to {\mathrm{Dye}}^*/{\mathrm{TiO}}_2$$

Electron injection:

2 $${\mathrm{Dye}}^*/{\mathrm{TiO}}_2 \to {\mathrm{Dye}}^{ \bullet + }/{\mathrm{TiO}}_2\left( {e^ - } \right)$$

Dye regeneration:

3 $${\mathrm{Dye}}^{ \bullet + }/{\mathrm{TiO}}_2\left( {e^ - } \right) + 2{\mathrm{I}} ^- \to {\mathrm{Dye}}/{\mathrm{TiO}}_2\left( {e^ - } \right) + \rm I_2^{ \bullet - }$$

Electrolyte recombination:

4 $${\mathrm{Dye}}^{ \bullet + }/{\mathrm{TiO}}_2\left( {e^ - } \right) + {\mathrm{I}}_3^ - \to {\mathrm{Dye}}^{ \bullet + }/{\mathrm{TiO}}_2 + {\mathrm{I}}_2^{ \bullet - } + {\mathrm{I}}^ - $$

Back-electron transfer (BET):

5 $${\mathrm{Dye}}^{ \bullet + }/{\mathrm{TiO}}_2(e^ - ) \to {\mathrm{Dye}}/{\mathrm{TiO}}_2$$

**Fig. 4:**
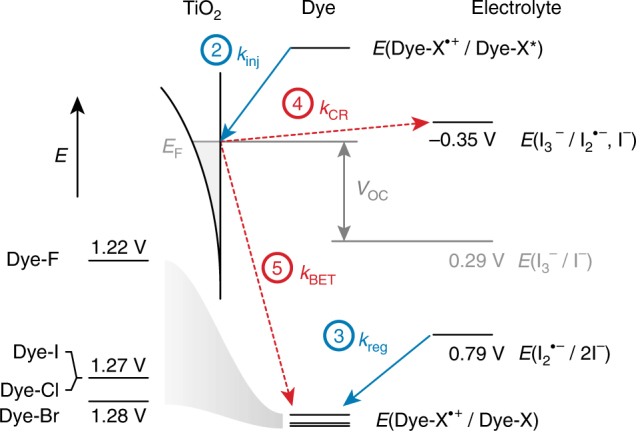
Landscape of energy levels. Summary of the ground-state and excited-state reduction potentials for the Dye-X series, relevant redox couples for an iodine-based electrolyte, and rate constants for electrolyte charge recombination (*k*_CR_), back-electron transfer (*k*_BET_), and dye regeneration (*k*_reg_). All potentials are reported in V vs. NHE. The numbered reactions correspond to Eqs. 1–5

Dye regeneration is widely assumed to be quantitative in the DSSC largely because of high incident photon-to-current efficiencies (IPCEs) and independent kinetics experiments that indicated dye regeneration to be much faster than electrolyte recombination and BET^30–35^. However, these experiments are not necessarily representative of the maximum power point of the device where a higher concentration of conduction band electrons affects the relative rates of the interfacial reactions, as in Eqs. (3–5)^3,16,36,37^. For example, the rate of BET may become competitive with dye regeneration at full illumination, thereby reducing the electron concentration in the TiO_2_ conduction band (${n_{TiO_2}}$), and, in turn, the open-circuit photovoltage (*V*_OC_)^3,37^. Any gains in the efficiency of the regenerative step (*η*_reg_; Eq. (6), where *x* represents the BET reaction order in TiO_2_(e^−^)^16^) will therefore reduce the rate of BET, which reduces the equilibrium concentration of oxidized dyes on the surface of the TiO_2_ susceptible to BET. This scenario increases the energy of the quasi-Fermi level and increases the *V*_OC_ and power conversion efficiency (PCE) of the cell (Fig. 4)^3^.

Regeneration efficiency:

6 $$\eta _{{\mathrm{reg}}} = \frac{{k_{{\mathrm{reg}}}\left[ {{\mathrm{I}}^ - } \right]}}{{k_{{\mathrm{reg}}}\left[ {{\mathrm{I}}^ - } \right] + k_{{\mathrm{BET}}} \times n_{{\mathrm{TiO}}_2}^x}}$$

**Fig. 5:**
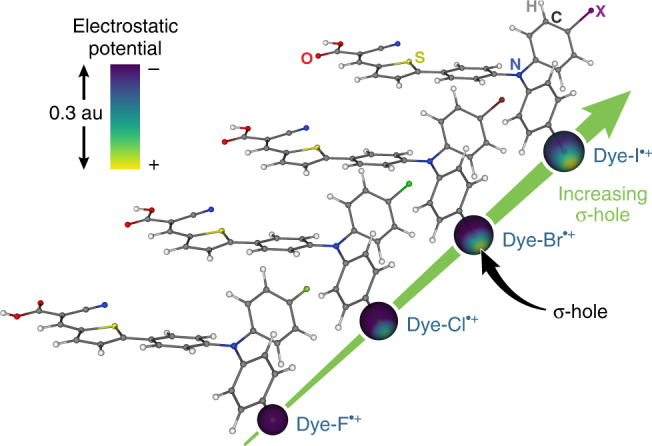
Increasing *σ*-hole on Dye-X series. DFT models of the singly-oxidized dyes, Dye-X^•+^ (where X is F, Cl, Br, and I), reveal an increasingly electropositive σ-hole on the terminus of the halogen substituents as the size of the halogen increases. The electrostatic potential is plotted over a sphere corresponding to the Van der Waals radius of the respective halogen substituent

**Fig. 6:**
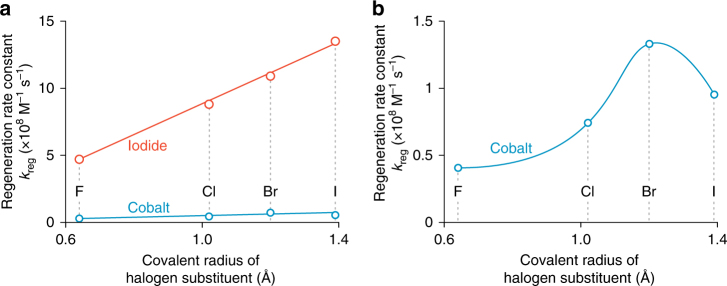
Regeneration rate is proportional to halogen polarizability. **a** Regeneration rate constants (*k*_reg_) for the reaction of Dye-X^•+^ (X is F, Cl, Br, I) on titanium dioxide (TiO_2_) substrates with 0.5–10 mM of I^−^ (red) or 10–150 mM of [Co(bpy)_3_]^2+^ (blue). Dye-X^•+^ is created through 532 nm photoinduced electron injection. **b** In the case of iodide, *k*_reg_ follows a linear correlation with substituent size, but the cobalt electrolyte tracks more closely with the Δ*G*_rxn_ for the reaction between Dye-X^•+^ and [Co(bpy)_3_]^2+^ (**b** and Fig. 1)^4,11^

Our interest in studying interfacial charge transfer chemistry led to our recent discovery that weak halogen bonding between the electrolyte and the dye may exist in the DSSC^4,11^. Previous nanosecond spectroscopic studies have shown that *k*_reg_ increases with increasing halogen substituent size for the series, Dye-F^•+^ < Dye-Cl^•+^ < Dye-Br^•+^ < Dye-I^•+^^4^. The congruent optical and thermodynamic properties of the four Dye-X compounds presented an effective platform to resolve differences in rates of dye regeneration by nucleophilic iodide: The *k*_reg_ values and *V*_OC_s were found to increase with the size of the halogen atoms on Dye-X, which in turn correlates to the size of the *σ*-hole (Fig. 5)^11^. This trend is consistent with interfacial halogen bonding between Dye-X^•+^ and iodide. These lines of evidence notwithstanding, we are unable to rule out the possibility that the effect is simply due to London dispersion forces arising from differences in Van der Waals radii of the halogen substituents (Fig. 6). The situation is further complicated by our inability to detect halogen bonding interactions between Dye-X and iodide by differences in optical spectra^14,25,38^.

The XAS data presented in this study confirms an interfacial halogen bond between chloride and Dye-Br^•+^ and Dye-I^•+^. Greater orbital overlap between the bromine substituents and chloride leads to a more significant halogen bond than that between chloride and Dye-I^•+^. In the DSSC where iodide is used, greater orbital overlap exists with Dye-I^•+^; a feature that is supported by higher *k*_reg_ values^11^. The direct detection of halogen bonding in this study provides direct experimental evidence that the reactive fragment of the oxidized dye is the *β*-LUSO. This unprecedented spatial resolution of the dye^+^–halide interaction is supported by several lines of experimental evidence, and provides a clear picture for dye design.

A less obvious picture that emerges from this study is that dye regeneration is enabled by the formation of a charge-assisted halogen bond—an intermolecular interaction that only exists for the oxidized dye. It is important to consider these results with the relevant stoichiometry of a DSSC. Each TiO_2_ nanocrystallite has approximately 500 dyes anchored to its surface, and the sensitized thin films are immersed in a 0.6 M iodide solution, such that the closest iodide is on average 8 Å away from any given dye (See Supplementary Discussion: Spacial calculations). Upon AM 1.5 solar illumination, equilibrium is no longer present and the concentrations of transient interfacial species reach a steady state. Each dye injects an electron into TiO_2_ about once per second and is regenerated on the 10^−3^–10^−6^ s timescale. At the point of maximum power conversion for the DSSC, there is about 1 oxidized dye present at any given time out of every 100,000 dyes, and an estimated 20–30 electrons are present in each nanocrystallite (See Supplementary Discussion: Temporal calculations)^3,39^. The electric field generated by these electrons is near 1.5 MV cm^−1^ and influences mass transport through migration of the anionic I^−^ away from the sensitized interfaces^40^. Halogen bonding occurs in opposition to this field and provides an inner-sphere pathway for sensitizer regeneration. The inner-sphere adduct provides strong electronic coupling through *β*-LUSO–iodide overlap that enhances regeneration and DSSC efficiency. Further improvements in DSSCs are expected if halogen bonding occurred with the ground-state dye molecules, thereby removing the diffusional component of dye regeneration completely. Studies to further investigate these effects are currently under way in our laboratories.

In conclusion, XAS was used to directly confirm an interfacial halogen bonding interaction between a soluble halide species and immobilized dyes bearing different halogen substituents. This work confirms that these intermolecular interactions only exist when the dyes bear polarizable halogen substituents like bromine and iodine, as opposed to non-polarizable substituents like fluorine, as expected from halogen bonding theory^12,13^. What is more interesting is that this work also demonstrates that these halogen bonding interactions occur after electron injection into the TiO_2_, and thus can be an important factor only during the short lifetime of the oxidized dye. Furthermore, we have demonstrated using computational methods that these halogen bonding interactions are not significantly affected by the identity of the nucleophilic halide species, and therefore we believe that these results presented here with chloride can be extended to the dye-iodide interactions that occur within DSSCs. As such, we can now confirm that the previously observed increases in dye regeneration rate by iodide^14^, and by extension *V*_OC_ and PCE in functional DSSCs^11^, can be definitively attributed to the increased halogen bond donating ability of Dye-Br^•+^ and Dye-I^•+^. It is particularly striking that such a weak intermolecular interaction involving a distinctively minority species can have such a huge effect on the measured photovoltage, and it suggests that encouraging other intermolecular interactions between oxidized dye and electrolyte species may have similar beneficial effects on dye performance.

## Methods

### Synthetic methods

The Dye-X series was prepared and characterized as previously described^4^.

### Physical methods

Acetonitrile (CH_3_CN) was purchased from Honeywell (Burdick and Jackson, 99.9%) and used as received. The Carbowax^TM^ employed, polyethyleneglycol bisphenol A epichlorohydrin copolymer (mol wt 15,000–20,000 Da), was purchased from Sigma Aldrich, and was ground by mortar and pestle prior to use to encourage dissolution. Kapton^TM^ polyimide tape (1″ × 3 m, silicone adhesion) was purchased from McMaster-Carr. Nitrosonium tetrafluoroborate (98%) was purchased from Alfa Aesar and was used as received. Concentrated nitric acid (reagent grade) was purchased from Fisher Scientific and used as received. Titanium(IV) isopropoxide (97%) was purchased from Sigma Aldrich and was used as received. Tetraethylammonium chloride (anhydrous) was prepared by drying tetraethylammonium chloride hydrate (98%, Alfa Products) by literature methods^41^.

### Device fabrication

The TiO_2_ nanoparticle sol-gel was prepared as previously described^42^. In short: 20 mL of deionized water and a stir bar were placed in a 125 mL Erlenmeyer flask, and the volume was marked. An additional 40 mL of deionized water was added for a total of 60 mL of deionized water. An aliquot of 0.42 mL of concentrated nitric acid was added. After proper mixing, the flask was covered with aluminum foil and 10 mL of titanium tetraisopropoxide was added dropwise over the course of 20 min while the solution was vigorously stirred. The flask was heated in a water bath while vigorously stirring at ~95 °C for 6 h, during which the precipitate dissolved resulting in a pale blue opaque solution. The solution was heated until 20 mL remained, resulting in a 150–170 g L^−1^ final concentration. The solution was placed in a Teflon acid digestion bomb and heated to 200 °C for 12 h. The final product appeared like a water-based glue with some liquid on top. While still warm, 1 g of ground carbowax was stirred into the solution. The solution was stored in a constantly stirred, foil-covered glass vial. After 24 h the solution was ready to use, and was kept for several months. These nanoparticles have been measured through SEM to be 10–20 nm in diameter.

### TiO_2_ slide preparation

Glass microscope slides were cut to size and drilled as depicted in Supplementary Fig. 1. The glass was cleaned with ethanol and methanol, and was allowed to air dry. Scotch^TM^ tape was used to mask out the area required. A small aliquot of the TiO_2_ solution was placed at one end of the masked out window, and a disposable, clean glass pipette was used to streak the TiO_2_ solution across the surface. The slide was covered and allowed to air dry for 30 min. The slide was heated to 450 °C for 30 min, and cooled to room temperature over 15 min. While the slides were still warm, they were placed into dry solutions of acetonitrile containing the relative Dye-X compound. The slides were allowed soak for 12 h, after which the titanium dioxide surface was saturated with dye. Finally, the slides were rinsed briefly with dry acetonitrile.

### Device assembly and preparation

The TiO_2_ surface of the dyed glass slides were covered with a layer of 0.2 mm 2520 polypropylene for XRF by ATS Scientific Inc, which acted as an X-ray-transparent window. The polypropylene was secured using sulfur-free Kapton^TM^ tape. To access the oxidized form of the dyes, the cavity above the sensitized surface of the device was washed with a concentrated solution of nitrosonium tetrafluoroborate in dry acetonitrile by flushing the solution through the two pre-drilled holes on the device immediately prior to use. The cavity was then flushed and filled with a 100 mM solution of tetrabutylammonium chloride in dry acetonitrile. See Supplementary Fig. 1 for details.

### XAS data collection

Chlorine K-edge XAS data was collected at beamline 14–3 at the Stanford Synchrotron Radiation Laboratory. The sample was analyzed at ambient temperatures and pressures in a helium environment. The beam is unfocused over a size of 1 mm × 6 mm with an energy resolution of ~1 × 10^−4^ ΔE E^−1^ with ring conditions of 3 GeV and 500 mA to allow for high energy-resolution measurements on homogeneous samples. Data points were taken at several points across the surface of the cell to ensure homogeneity.

### XAS data analysis

SixPack was used to calibrate and average all acceptable spectra^43^. NaCl (*E*_0_ = 2820.2 eV^19^) spectra acquired at the same time as sample data were used as a calibrant for Cl K-edge spectra. BlueprintXAS version 2.7 was then used for background subtraction and normalization. To minimize the user bias introduced during data work-up, BlueprintXAS fits the spline, peaks, and background concurrently^27,44^, while the parameters for each variable are user defined. (Supplementary Tables 1–7.) The fits were run in AUTO mode as in this mode, a Monte Carlo methodology is used to choose the starting point of each fit to further remove user bias. Each fit contained the following components: edge peak fit; spline + edge peak fit; pre-edge peak fit. General method for setting parameter restrictions in each of the fits:

- *E*_0_ range/spline lower limit: energy of the edge peak +5 eV
- Peak intensity ranges (I1 and I2): limits must be sufficiently large to avoid truncating solution distribution
- Energy of peak: ±1–2 eV from visual peak maximum
- % Gaussian contribution to shape of peak (G#): 100%
- Background lower limit: ~20 eV range below *E*_0_

### Computational methods

Quantum-mechanical calculations were run using the Gaussian 09 (G09) computational package^45^, or the ORCA 3.0.3 computational package^46^, as indicated. All geometry optimizations were performed in G09 using the unrestricted M06-2X functional with a conductor-like polarizable continuum solvation model (CPCM) of acetonitrile; an ultrafine integration grid (99 radial shells, 590 angular points); and the aug-cc-pVDZ-PP basis set on bromine and iodine, aug-cc-pVDZ on fluorine and chlorine, and cc-pVDZ on all other atoms^46–52^. All geometries were optimized to a minimum (Supplementary Fig. 5) and frequency calculations performed at the same level of theory to verify the absence of imaginary frequencies, except where noted. To ensure that the charges were distributed appropriately during the optimization of the interacting ion pairs, an initial guess was generated specifying a negative charge on the halide, and this fragmented guess was read in as the initial guess for the optimization and frequency calculations. Subsequent calculations were performed using one of two different methodologies:

*Method A*: Single point energies for determining the interaction energies, natural bond orbital analysis, and counterpoise corrections were calculated using identical parameters to the optimizations, except the aug-cc-pVTZ-PP basis set was used on bromine and iodine, aug-cc-pVTZ on fluorine and chlorine, and cc-pVTZ on all other atoms^47–53^.

*Method B*: Additional single point energies for determining interaction energies, chloride atomic orbital contributions to the halogen bonding MOs, and TD-DFT models of the pre-edge feature were calculated in ORCA using the B3LYP functional with G3 dispersion correction^53–57^, the def2-TZVP all-electron basis set on all atoms^58,59^, the zero-order regular approximation^59–63^, and a refined integration grid (Grid6: Lebedev = 590, IntAcc = 5.34). These calculations also made use of the libint2 library for the determination of 2-electron integrals^64^.

Parameters for the aug-cc-pVDZ-PP and aug-cc-pVTZ-PP basis sets were obtained from the ESML basis set exchange^65,66^. The interaction energies Δ*E*_int_) were estimated using the following formula:

7 $$\Delta E_{{\mathrm{int}}} = E_{{\mathrm{complex}}}-(E_{{\mathrm{Dye}}} + E_{{\mathrm{halide}}})$$

Where *E*_Dye_ is the single point energy of the isolated dye molecules; *E*_halide_ is the single point energy of the halide (*E*_chloride_ = −460.3847105 a.u. with method A, *E*_chloride_ = −462.5113703 a.u. with method B); and *E*_complex_ is the energy of the interacting pairs. For comparison, an analogous analysis was performed on the interaction pairs with iodide (*E*_iodide_ = −295.8129275 a.u. with method A, *E*_iodide_ = −7224.172175 a.u. with method B). *E*_complex_ values for these iodide interactions with oxidized Dye-X^•+^ were previously reported^11^, while the values for interaction with neutral Dye-X were recalculated from previously reported models with updated basis sets to match the methods used in this study^4^. *E*_complex_ values calculated with method A were corrected for basis set superposition error (BSSE) using the counterpoise method in the gas phase;^66–69^ however this correction was not applied to *E*_complex_ values calculated with method B. Given the larger absolute value of Δ*E*_int_ obtained using method B, BSSE is expected to have a considerably smaller proportional impact on these energies and is not expected to affect the overall trend in the data. Natural bond orbital (NBO) analyses of intermolecular interactions were performed with the NBO 6.0 program^70^ (Supplementary Tables 11–18). Molecular orbitals were visualized at a constant iso value of 0.03 using the Jmol software package.

### Data availability

XAS and DFT data that support the findings of this study have been deposited GitHub with the identifier DOI: 10.5281/zenodo.844914 (https://github.com/berlinguette/Parlane2017).

## Electronic supplementary material

Supplementary Information
