# Supplementary material for: Spectroscopic detection of halogen bonding resolves dye regeneration in the dye-sensitized solar cell
Source: Nat Commun. 2017 Nov 24;8:1761. doi: 10.1038/s41467-017-01726-7 (PMC5701207; doi:10.1038/s41467-017-01726-7)
Supplement: Supplementary file 1 — Supplementary Information [file 41467_2017_1726_MOESM1_ESM.pdf]

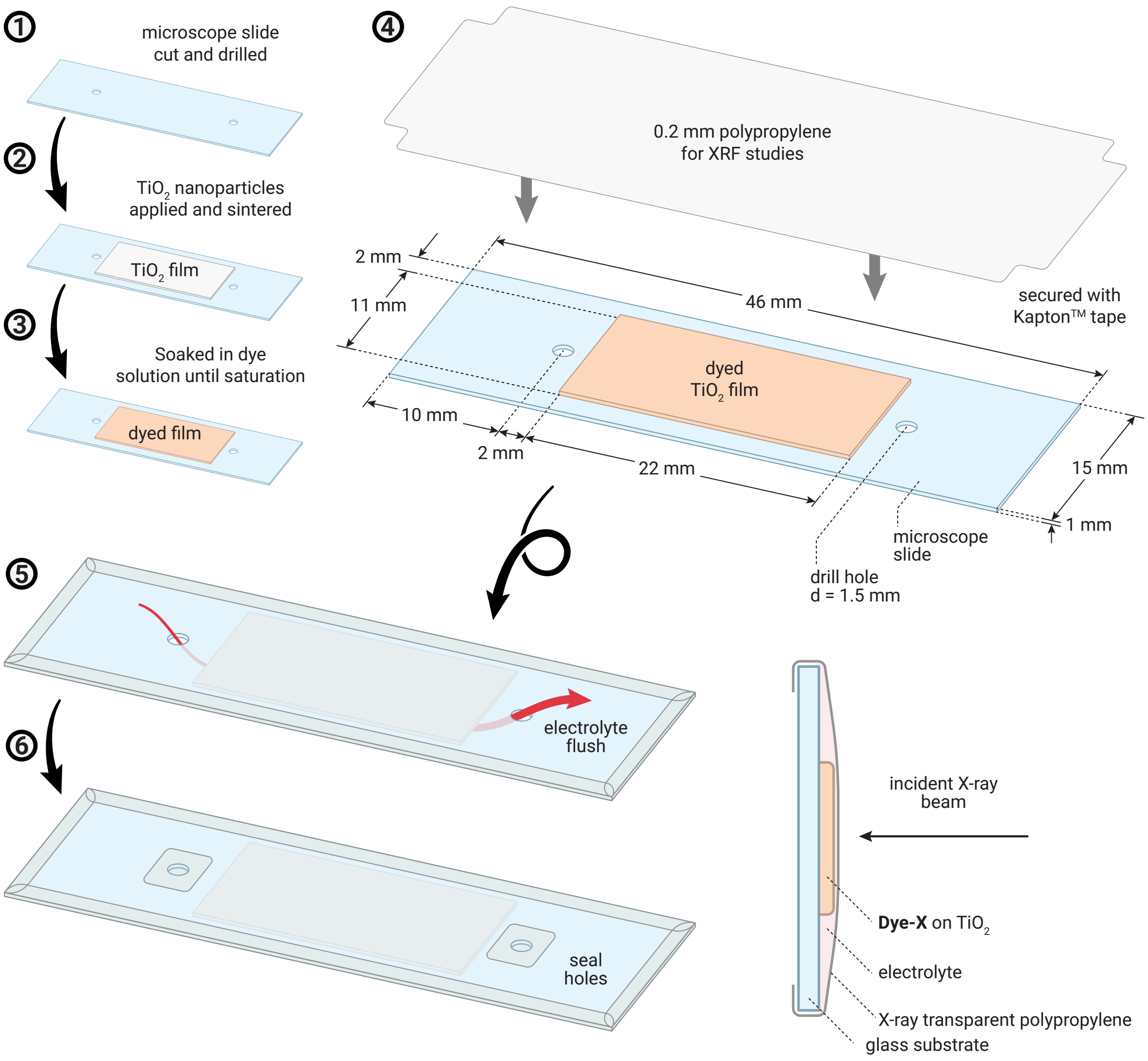

Supplementary Figure 1: **Slide construction.** Microscope slides were 1) cut and drilled to size, 2) sintered with titanium dioxide nanoparticles, 3) soaked in a dying solution, 4) sealed with a X-ray transparent polypropylene window, 5) flushed with oxidant and electrolyte solutions, and 6) sealed with Kapton^{TM} tape.

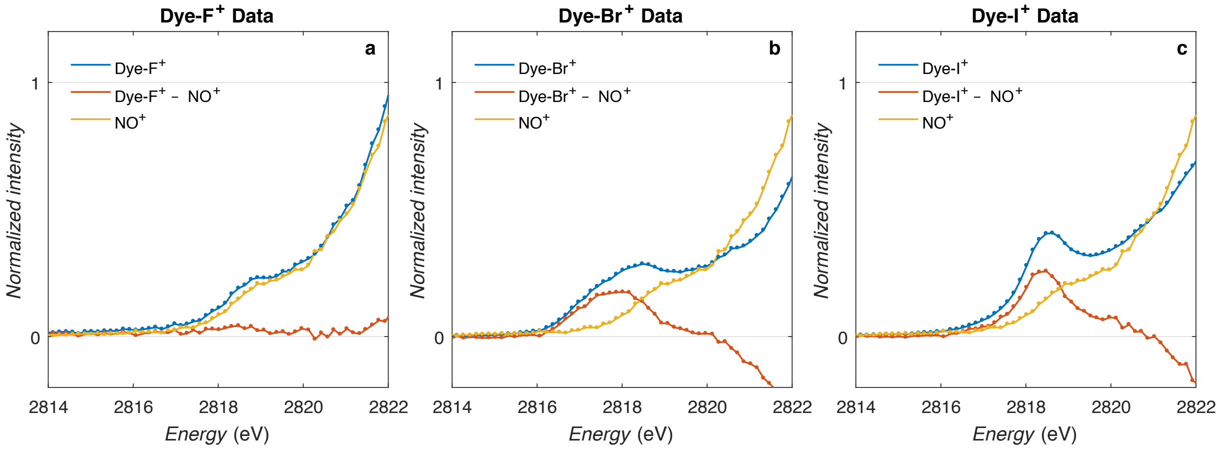

Supplementary Figure 2: **Background Correction of the XAS pre-edge feature in the Dye-X$^{\bullet+}\cdots$Cl^{-} series.** Chlorine K-edge XAS spectra of the Dye-X$^{\bullet+}\cdots$Cl^{-} series. The normalized control spectra (yellow) was subtracted from the normalized experimental spectra (blue), affording a qualitative comparison of the relative contributions of the $\sigma^*_{\mathrm{XB}} \leftarrow \mathrm{Cl}_{1s}$ transition to the XAS pre-edge feature (red).

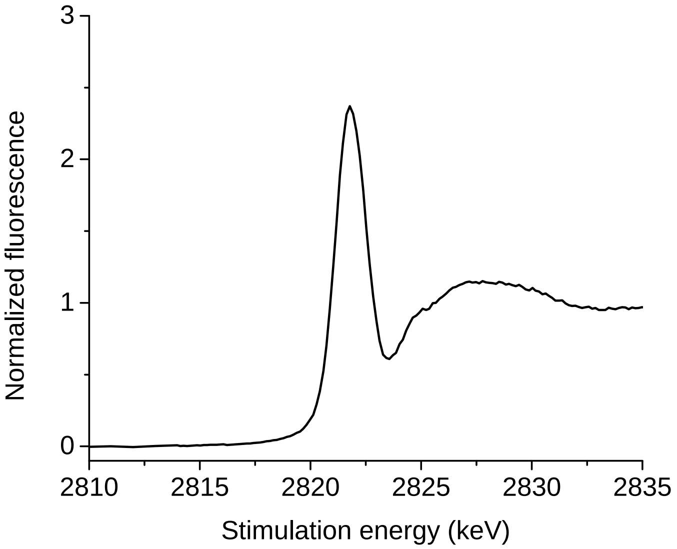

Supplementary Figure 3: **Cl K-edge of Dye-Cl$\cdots$Cl$^-$.** Chlorine K-edge XAS spectra of the Dye-Cl$\cdots$Cl$^-$ sample, showing an intense contribution from the covalently-bound chlorine swamping the signal from the chloride.

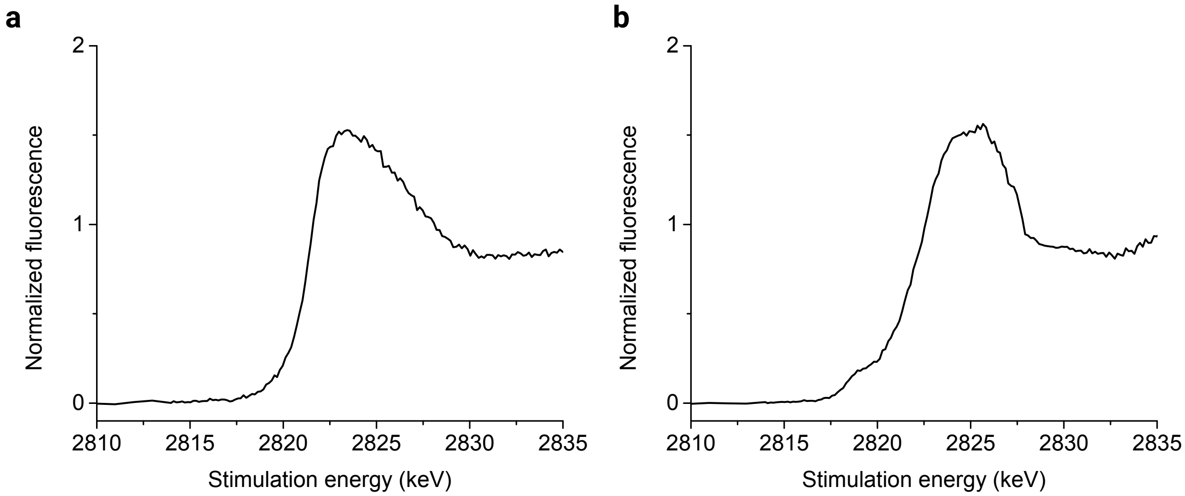

Supplementary Figure 4: **Cl K-edge of tetrabutylammonium chloride.** Chlorine K-edge XAS spectra of a) a 100 mM solution of tetrabutylammonium chloride in dry acetonitrile, and b) a 100 mM solution of tetrabutylammonium chloride in dry acetonitrile saturated with nitrosonium tetrafluoroborate.

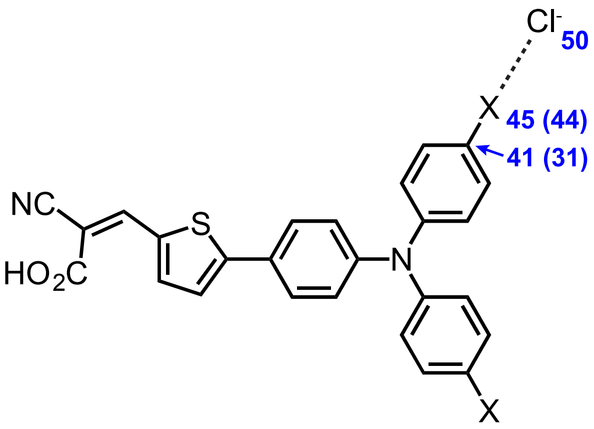

Supplementary Figure 5: **Dye-X$\cdots$Cl$^-$ interaction geometries.** Schematic representation of the Dye-X$\cdots$Cl$^-$ interaction geometries. Selected atom numbers used in NBO analysis are listed in blue; numbers in parentheses apply to Dye-Br and Dye-Br$^{\bullet+}$ only.

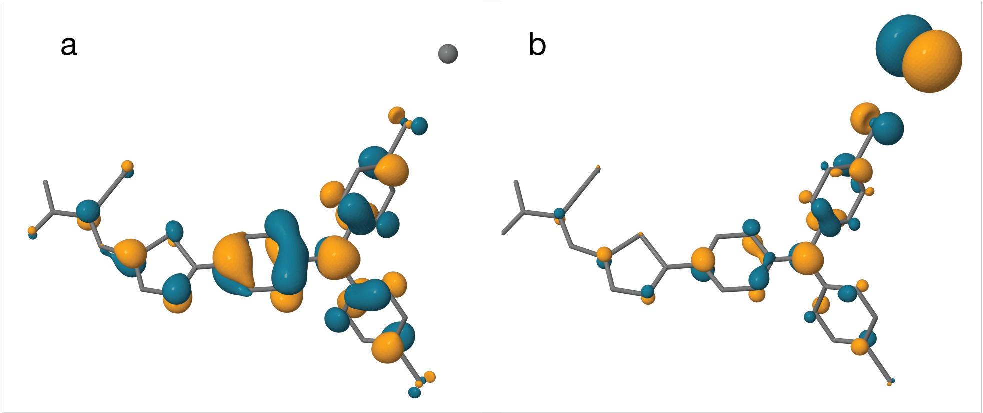

Supplementary Figure 6: **DFT functional effects on the β-LUSO of Dye-Br$\cdots$Cl$^-$.** β-LUSO plots at iso = 0.03 showing the calculated location of the positive hole on Dye-Br$^{\bullet+}\cdots$Cl$^-$. (a) Generated using DFT method A, which features a functional with 54% Hartree-Fock exchange, diffuse basis set functions included on halogen atoms, and acetonitrile solvent modeled with CPCM. (b) Plot was generated using DFT method B, which features a functional with 20% Hartree-Fock exchange, no diffuse functions, and no solvent model.

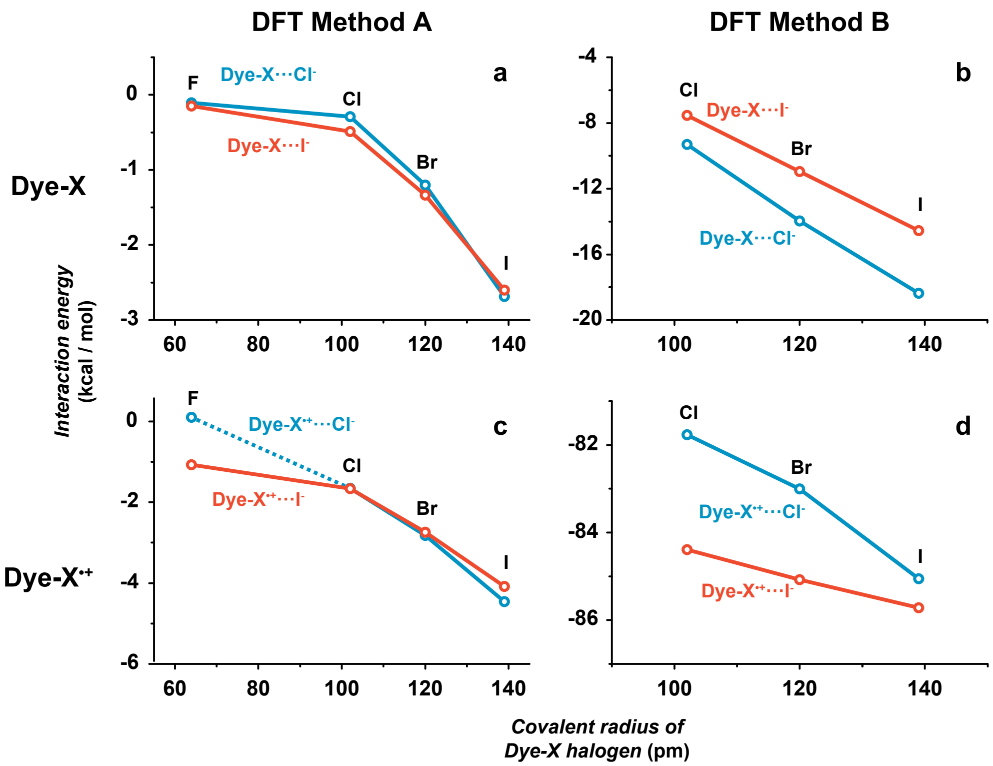

Supplementary Figure 7: **Stabilization energies of Dye-X···X⁻.** The interaction energies of the Dye-X series (above) and Dye-$X^{\bullet+}$ series (below) plotted as a function of the covalent radii of the halogen substituents. These values were calculated using either DFT method A (a, c) or method B (b, d). Note that the interaction energies calculated with method B were not corrected for BSSE. In addition, the geometry optimization for the Dye-$F^{\bullet+}$···Cl^- interaction was unable to find an energetic minimum, and therefore the chloride was arbitrarily placed at the van der Waals contact distance with the dye.

Supplementary Table 1: XAS results

Experiment	Normalized XAS Pre-edge feature	XAS Pre-edge feature energy (eV)	Experimental contribution of Cl_{3p} in XB*
**NO$^{\bullet+}\cdots$Cl$^-$**	0.13±0.02 %	2818.6	Not applicable
**Dye-I$\cdots$Cl$^-$**	No contribution	No contribution	No contribution
**Dye-F$^{\bullet+}\cdots$Cl$^-$**	0.21±0.06 %	2818.6	3.5 %
**Dye-Br$^{\bullet+}\cdots$Cl$^-$**	0.59±0.03 %	2818.3	7.7 %
**Dye-I$^{\bullet+}\cdots$Cl$^-$**	0.43±0.02 %	2818.5	5.6 %

Supplementary Table 2: XAS fit parameters for series

Experiment	Total fits	SSE	r^2	fy-Projection
**NO$^{\bullet+}\cdots$Cl$^-$**	71	1.2312E-04	0.99599	0.999497
**Dye-I$\cdots$Cl$^-$**	80	6.3177E-06	0.979782	0.998803
**Dye-F$^{\bullet+}\cdots$Cl$^-$**	70	1.2318E-04	0.996161	0.999497
**Dye-Br$^{\bullet+}\cdots$Cl$^-$**	82	9.6398E-04	0.992981	0.999025
**Dye-I$^{\bullet+}\cdots$Cl$^-$**	89	2.1368E-04	0.995682	0.999508

Supplementary Table 3: XAS fit parameters for Dye-I$\cdots$Cl$^-$

Parameter	ID	Average	S.D.
**Spline & edge peak fitting**			
Degree-Pre-edge	M1	2.4968E+00	9E-01
Degree-Spline1	N1	2.4997E+00	8E-01
E0 (edge-Spline)	E0	2.8273E+03	7E-01
hwhm	W1	5.4215E+00	5E-01
Pre-edge, Max	B1	2.8115E+03	4E+00
Spline, Min	C1	2.8249E+03	3E+00
Intensity	I1	2.1889E-02	1E-03
Position	O1	2.8241E+03	2E-01
hwhm	W2	2.9693E+00	8E-02
Gaussian, fraction	G2	6.0000E-01	1E-14

Supplementary Table 4: XAS fit parameters forNO$^{\bullet+}\cdots$Cl^{-}

Parameter	ID	Average	S.D.
**Spline & edge peak fitting**			
Degree-Pre-edge	M1	2.4310E+00	9E-01
Degree-Spline1	N1	2.5626E+00	8E-01
E0 (edge-Spline)	E0	2.8269E+03	7E-01
hwhm	W1	4.5471E+00	7E-01
Pre-edge, Max	B1	2.8110E+03	3E+00
Spline, Min	C1	2.8279E+03	1E+00
Intensity	I1	1.8292E-01	1E-02
Position	O1	2.8247E+03	6E-02
hwhm	W2	2.7832E+00	5E-02
Gaussian, fraction	G2	9.2093E-01	7E-02
**Pre-edge peak fitting**			
Intensity	I2	2.6848E-03	4E-04
Position	O2	2.8187E+03	1E-12
hwhm	W3	7.2249E-01	4E-02
Gaussian, fraction	G3	9.7674E-01	1E-01

Supplementary Table 5: XAS fit parameters forDye-F$^{\bullet+}\cdots$Cl^{-}

Parameter	ID	Average	S.D.
**Spline & edge peak fitting**			
Degree-Pre-edge	M1	2.3924E+00	9E-01
Degree-Spline1	N1	2.4872E+00	9E-01
E0 (edge-Spline)	E0	2.8256E+03	7E-01
hwhm	W1	4.5824E+00	7E-01
Pre-edge, Max	B1	2.8109E+03	3E+00
Spline, Min	C1	2.8273E+03	2E+00
Intensity	I1	1.3059E-01	5E-03
Position	O1	2.8244E+03	7E-02
hwhm	W2	2.5005E+00	3E-02
Gaussian, fraction	G2	9.6977E-01	6E-02
**Pre-edge peak fitting**			
Intensity	I2	4.7062E-03	1E-03
Position	O2	2.8187E+03	3E-12
hwhm	W3	1.0000E+00	9E-15
Gaussian, fraction	G3	6.9116E-01	3E-01

Supplementary Table 6: XAS fit parameters forDye-Br$^{\bullet+}\cdots$Cl^{-}

Parameter	ID	Average	S.D.
**Spline & edge peak fitting**			
Degree-Pre-edge	M1	2.3458E+00	9E-01
Degree-Spline1	N1	2.6265E+00	9E-01
E0 (edge-Spline)	E0	2.8254E+03	8E-01
hwhm	W1	4.7677E+00	2E-01
Pre-edge, Max	B1	2.8103E+03	3E+00
Spline, Min	C1	2.8272E+03	1E+00
Intensity	I1	3.2567E-01	2E-02
Position	O1	2.8246E+03	2E-02
hwhm	W2	2.0659E+00	5E-02
Gaussian, fraction	G2	6.0000E-01	9E-15
**Pre-edge peak fitting**			
Intensity	I2	2.8480E-02	2E-03
Position	O2	2.8184E+03	3E-02
hwhm	W3	1.2394E+00	3E-02
Gaussian, fraction	G3	6.0000E-01	2E-12

Supplementary Table 7: XAS fit parameters forDye-I$^{\bullet+}\cdots$Cl^{-}

Parameter	ID	Average	S.D.
**Spline & edge peak fitting**			
Degree-Pre-edge	M1	2.5632E+00	9E-01
Degree-Spline1	N1	2.2918E+00	8E-01
E0 (edge-Spline)	E0	2.8248E+03	2E-01
hwhm	W1	5.7101E+00	3E-01
Pre-edge, Max	B1	2.8112E+03	3E+00
Spline, Min	C1	2.8274E+03	2E+00
Intensity	I1	1.7895E-01	1E-02
Position	O1	2.8246E+03	2E-02
hwhm	W2	2.3880E+00	4E-02
Gaussian, fraction	G2	4.5766E-01	7E-02
**Pre-edge peak fitting**			
Intensity	I2	1.2799E-02	8E-04
Position	O2	2.8186E+03	3E-03
hwhm	W3	6.8019E-01	2E-02
Gaussian, fraction	G3	4.0000E-01	2E-12

Supplementary Table 8: Calculated optimum halogen-chloride distances ($d_{X\leftarrow Cl^-}$); van der Waals distances (d_{vdW}); interaction energies (ΔE_{int}); and chloride charge. Calculations were performed with method A.

Compound	$d_{X\leftarrow Cl^-}$ (Å)	d_{vdW} (Å)[a]	ΔE_{int} (kcal mol^{-1})	Chloride charge[b]
**Dye-F**	4.25	3.28	-0.11	-1.000
**Dye-Cl**	3.49	3.56	-0.29	-0.993
**Dye-Br**	3.37	3.66	-1.20	-0.982
**Dye-I**	3.36	3.79	-2.69	-0.967
**Dye-F$^{\bullet+}$**, [c]	3.28	3.28	0.10	-0.999
**Dye-Cl$^{\bullet+}$**	3.39	3.56	-1.66	-0.990
**Dye-Br$^{\bullet+}$**	3.29	3.66	-2.82	-0.977
**Dye-I$^{\bullet+}$**	3.27	3.79	-4.46	-0.955

[a]Defined as the sum of the halogen van der Waals distance plus the Pauling ionic radius of chloride, as appropriate. [b]Charge determined using natural population analysis as a part of NBO analysis. [c]No energetic minimum was found during the geometry optimization for this interaction pair, therefore chloride was arbitrarily placed at the van der Waals distance from the dye.

Supplementary Table 9: Key second order Dye-X$\cdots$Cl$^-$ / Dye-X$^{\bullet+}\cdots$Cl$^-$ interactions based on optimized halogen-chloride interaction structures (Figure 5). Stabilization energy (E^2) values and F_{ij} values are reported as the sum of alpha and beta terms for equivalent interactions; ϵ_j-ϵ_i values are reported as the average of alpha and beta terms. Calculations were performed with method A.

Compound	Donor	Acceptor	Donor occupancy	E^2 (kcal mol^{-1})	F_{ij} (a.u.)	ϵ_j-ϵ_i (a.u.)
**Dye-F**	60α. LP(4) Cl50<br>60β. LP(4) Cl50	194α. BD*(1) C41-F45<br>194β. BD*(1) C41-F45	1.99992	none	none	none
**Dye-Cl**	69α. LP(4) Cl50<br>69β. LP(4) Cl50	202α. BD*(1) C41-Cl45<br>202β. BD*(1) C41-Cl45	1.99270	1.50	0.050	0.53
**Dye-Br**	77α. LP(4) Cl50<br>77β. LP(4) Cl50	196α. BD*(1) C31-Br44<br>196β. BD*(1) C31-Br44	1.98218	3.56	0.074	0.48
**Dye-I**	77α. LP(4) Cl50<br>77β. LP(4) Cl50	210α. BD*(1) C41-I45<br>210β. BD*(1) C41-I45	1.96748	5.88	0.092	0.46
**Dye-F$^{\bullet+}$**, [a]	61α. LP(4) Cl50<br>59β. LP(4) Cl50	194α. BD*(1) C41-F45<br>194β. BD*(1) C41-F45	1.99858	0.46	0.032	0.66
**Dye-Cl$^{\bullet+}$**	71α. LP(4) Cl50<br>67β. LP(4) Cl50	202α. BD*(1) C41-Cl45<br>202β. BD*(1) C41-Cl45	1.99003	1.99	0.058	0.52
**Dye-Br$^{\bullet+}$**	77α. LP(4) Cl50<br>75β. LP(4) Cl50	196α. BD*(1) C31-Br44<br>196β. BD*(1) C31-Br44	1.97723	4.40	0.082	0.48
**Dye-I$^{\bullet+}$**	78α. LP(4) Cl50<br>75β. LP(4) Cl50	210α. BD*(1) C41-I45<br>210β. BD*(1) C41-I45	1.95657	7.65	0.106	0.46

[a]No energetic minimum was found during the geometry optimization for this interaction pair, therefore chloride was arbitrarily placed at the van der Waals distance from the dye.

Supplementary Table 10: DFT interaction energies for Dye-X / Dye-X$^{\bullet+}$, [a] series

		Interaction Energy (kcal / mol)			
		Method A		Method B	
**X$^-$**	=	Cl$^-$	I$^-$	Cl$^-$	I$^-$
Dye-F···X$^-$		-0.1068	-0.1492	N/A	N/A
Dye-Cl···X$^-$		-0.2899	-0.4873	-9.3022	-7.5412
Dye-Br···X$^-$		-1.2048	-1.3374	-13.9634	-10.9604
Dye-I···X$^-$		-2.6877	-2.6001	-18.3770	-14.5553
Dye-F$^{\bullet+}$···X$^-$		0.0979	-1.0705	N/A	N/A
Dye-Cl$^{\bullet+}$···X$^-$		-1.6567	-1.6665	-81.7670	-84.3938
Dye-Br$^{\bullet+}$···X$^-$		-2.8185	-2.7436	-83.0071	-85.0781
Dye-I$^{\bullet+}$···X$^-$		-4.4610	-4.0867	-85.0552	-85.7187

[a]No energetic minimum was found during the geometry optimization for the Dye-F$^{\bullet+}$···Cl$^-$ interaction pair, therefore chloride was arbitrarily placed at the van der Waals distance from the dye.

*Only orbital coefficients greater than 1.00% are reported.

Supplementary Table 11: Hybridization and Polarization Data for Selected NBOs of Dye-F···Cl$^-$

NBO	Orbital contributions
60α	LP(4) Cl50: 100% Cl50 (99.65% p)
60β	LP(4) Cl50: 100% Cl50 (99.65% p)
194α	BD*(1) C41-F45: 72.36% C41 (22.35% s, 77.46% p); 27.64% F45 (29.44% s, 70.29% p)
194β	BD*(1) C41-F45: 72.36% C41 (22.35% s, 77.46% p); 27.64% F45 (29.44% s, 70.29% p)

Supplementary Table 12: Hybridization and Polarization Data for Selected NBOs of Dye-Cl···Cl$^-$

NBO	Orbital contributions
69α	LP(4) Cl50: 100% Cl50 (4.06% s, 95.94% p)
69β	LP(4) Cl50: 100% Cl50 (4.06% s, 95.94% p)
202α	BD*(1) C41-Cl45: 55.19% C41 (22.99% s, 76.78% p); 44.81% Cl45 (17.90% s, 81.37% p)
202β	BD*(1) C41-Cl45: 55.19% C41 (22.99% s, 76.78% p); 44.81% Cl45 (17.90% s, 81.37% p)

Supplementary Table 13: Hybridization and Polarization Data for Selected NBOs of Dye-Br···Cl$^-$

NBO	Orbital contributions
77α	LP(4) Cl50: 100% Cl50 (5.85% s, 94.15% p)
77β	LP(4) Cl50: 100% Cl50 (5.85% s, 94.15% p)
196α	BD*(1) C31-Br44: 50.91% C31 (22.38% s, 77.35% p); 49.09% Br44 (12.91% s, 86.60% p)
196β	BD*(1) C31-Br44: 50.91% C31 (22.38% s, 77.35% p); 49.09% Br44 (12.91% s, 86.60% p)

Supplementary Table 14: Hybridization and Polarization Data for Selected NBOs of Dye-I$\cdots$Cl$^-$

NBO	Orbital contributions
77α	LP(4) Cl50: 100% Cl50 (7.33% s, 92.67% p)
77β	LP(4) Cl50: 100% Cl50 (7.33% s, 92.67% p)
210α	BD*(1) C41-I45: 44.57% C41 (22.26% s, 77.46% p); 55.43% I45 (9.50% s, 90.09% p)
210β	BD*(1) C41-I45: 44.57% C41 (22.26% s, 77.46% p); 55.43% I45 (9.50% s, 90.09% p)

Supplementary Table 15: Hybridization and Polarization Data for Selected NBOs of Dye-F$^{\bullet+}\cdots$Cl$^-$

NBO	Orbital contributions
61α	LP(4) Cl50: 100% Cl50 (3.30% s, 96.70% p)
59β	LP(4) Cl50: 100% Cl50 (3.31% s, 96.69% p)
194α	BD*(1) C41-F45: 71.80% C41 (22.83% s, 77.00% p); 28.20% F45 (29.04% s, 70.65% p)
194β	BD*(1) C41-F45: 72.02% C41 (22.87% s, 76.95% p); 27.98% F45 (29.35% s, 70.34% p)

Supplementary Table 16: Hybridization and Polarization Data for Selected NBOs of Dye-Cl$^{\bullet+}\cdots$Cl$^-$

NBO	Orbital contributions
71α	LP(4) Cl50: 100% Cl50 (4.34% s, 95.66% p)
67β	LP(4) Cl50: 100% Cl50 (4.37% s, 95.63% p)
202α	BD*(1) C41-Cl45: 54.05% C41 (23.68% s, 76.10% p); 45.95% Cl45 (17.58% s, 81.64% p)
202β	BD*(1) C41-Cl45: 54.44% C41 (23.68% s, 76.10% p); 45.56% Cl45 (17.77% s, 81.45% p)

Supplementary Table 17: Hybridization and Polarization Data for Selected NBOs of Dye-Br$^{\bullet+}\cdots$Cl$^-$

NBO	Orbital contributions
77α	LP(4) Cl50: 100% Cl50 (5.91% s, 94.09% p)
75β	LP(4) Cl50: 100% Cl50 (5.95% s, 94.15% p)
196α	BD*(1) C31-Br44: 49.45% C31 (23.14% s, 76.60% p); 50.55% Br44 (12.47% s, 86.98% p)
196β	BD*(1) C31-Br44: 49.94% C31 (23.12% s, 76.61% p); 50.06% Br44 (12.66% s, 86.79% p)

Supplementary Table 18: Hybridization and Polarization Data for Selected NBOs of Dye-I$^{\bullet+}\cdots$Cl$^-$

NBO	Orbital contributions
78α	LP(4) Cl50: 100% Cl50 (7.47% s, 92.53% p)
75β	LP(4) Cl50: 100% Cl50 (7.53% s, 92.47% p)
210α	BD*(1) C41-I45: 42.47% C41 (23.19% s, 76.55% p); 57.53% I45 (8.81% s, 90.72% p)
210β	BD*(1) C41-I45: 43.17% C41 (23.14% s, 76.59% p); 56.83% I45 (9.00% s, 90.53% p)

O'Regan et al. have shown that $\leq 1/100{,}000$ dyes are in the oxidized state for N719 under 1 sun illumination, based on the fact that a dye only injects once per second and regeneration times are ≤ 10 μs.[1] For the Dye-X series, we can confidently say that the regeneration rate for Dye-X is significantly faster than 10 μs (Figure 3). We can also show that it is quite reasonable that the Dye-X series would inject an electron once per second.

$$\begin{aligned}
&\textit{Since } j_{\mathrm{MP}} = 1.0642 \times 10^{-2}\mathrm{A\ cm^{-2}} \\
&\textit{and } \Gamma_{\mathrm{Dye}} = 10^{-7}\ \mathrm{mol\ cm^{-2}} \\
&\textit{and } \mathrm{C} = 6.242 \times 10^{18}\ \mathrm{A\ s} \\
&\textit{and } N_{\mathrm{A}} = 6.022 \times 10^{23}\ \mathrm{dyes\ mol^{-1}}
\end{aligned}
\qquad
\begin{aligned}
\textit{then } \Phi_{\mathrm{Dye}} &= \frac{j_{\mathrm{MP}}}{\Gamma_{\mathrm{Dye}}} \times \frac{\mathrm{C}}{N_{\mathrm{A}}} \\
&= \frac{(1.0642 \times 10^{-2}\mathrm{A\ cm^{-2}})}{(10^{-7}\ \mathrm{mol\ cm^{-2}})} \times \frac{(6.242 \times 10^{18}\ \mathrm{A\ s})}{(6.022 \times 10^{23}\ \mathrm{dyes\ mol^{-1}})} \\
&= 0.907\ \mathrm{electrons\ s^{-1}},\ \text{or} \\
\tau_{\mathrm{Dye}} &= \Phi_{\mathrm{Dye}}^{-1} \\
\tau_{\mathrm{Dye}} &= 1.10\ \mathrm{s}
\end{aligned}
\tag{1}$$

Therefore, claiming that $\leq 1/100{,}000$ dyes of the Dye-X series are oxidized at any given moment under operating conditions is a conservative estimate of the scarcity of the oxidized dyes under full illumination.

$$\begin{aligned}
\langle r \rangle &= a\Gamma\left(\frac{4}{3}\right) \\
\textit{where } a &= \left(\frac{3}{4\pi n}\right)^{\frac{1}{3}} \\
\textit{where } n &= \frac{N}{V}
\end{aligned}
\tag{2}$$

where $\langle r \rangle$ is the root-mean inter-particle distance for a randomly distributed population N in a given volume V, and where Γ is the Gamma function. Since

$$\begin{aligned}
n &= 3.61 \times 10^{26}\ \mathrm{m^{-3}}\ (\text{for } 0.6\ \mathrm{M\ I^{-}}) \\
a &= 8.71 \times 10^{-10}\ \mathrm{m} \\
\langle r \rangle &= 7.78 \times 10^{-10}\ \mathrm{m}\ \textit{or}\ 7.78\ \text{Å}.
\end{aligned}$$

Further, the root-mean-square displacement of a diffusing particle in three dimensions is given by

$$r_{rms} = \sqrt{6Dt} \tag{3}$$

Where r_{rms} is the root-mean-square displacement, D is the diffusion constant, and t is the timescale.

$$\begin{aligned} \textit{Since } D &= 2.72 \times 10^{-9}\ \mathrm{m}^2\ \mathrm{s}^{-1} \text{ for iodide in acetonitrile,} \\ \textit{and } t &= 10^{-9}\ \mathrm{s} \text{ for the average lifetime of Dye-I}^{+}, \\ r_{rms} &= \sqrt{6 \times (2.72 \times 10^{-9}\ \mathrm{m}^2\ \mathrm{s}^{-1}) \times (10^{-9}\ \mathrm{s})} \\ r_{rms} &= 4.04 \times 10^{-9}\ \mathrm{m} \textit{ or } 40.4\ \text{Å}. \end{aligned} \tag{4}$$

Therefore, since $r_{rms} > \langle r \rangle$, it is within the realm of possibility that an iodide could diffuse into close proximity with an oxidized dye within the lifetime of the oxidized dye.

1 Supplementary References

1. O'Regan, B. C. & Durrant, J. R. Kinetic and energetic paradigms for dyesensitized solar cells: Moving from the ideal to the real. *Acc. Chem. Res.* **42,** 1799–1808 (2009).
